# Publisher Correction: Sonogenetic control of mammalian cells using exogenous transient receptor potential A1 channels

**DOI:** 10.1038/s41467-022-28838-z

**Published:** 2022-02-25

**Authors:** Marc Duque, Corinne A. Lee-Kubli, Yusuf Tufail, Uri Magaram, Janki Patel, Ahana Chakraborty, Jose Mendoza Lopez, Eric Edsinger, Aditya Vasan, Rani Shiao, Connor Weiss, James Friend, Sreekanth H. Chalasani

**Affiliations:** 1 Molecular Neurobiology Laboratory, The Salk Institute for Biological Studies, La Jolla, CA 92037 USA; 2 Neurosciences Graduate Program, University of California San Diego, La Jolla, CA 92093 USA; 3 Medically Advanced Devices Laboratory, Department of Mechanical and Aerospace Engineering, Jacobs School of Engineering, University of California San Diego, La Jolla, CA 92093 USA

**Keywords:** Genetic techniques, Neural circuits, Electrophysiology

Correction to: *Nature Communications* 10.1038/s41467-022-28205-y, published online 9 February 2022.

The original version of this Article contained an error in Fig. 4b, in which the figure labels ‘Pre-US’ and ‘Post-US’ were incorrectly positioned above the label ‘Ultrasound 2.5 Mpa 100 ms’ with right alignment to the figure panels, instead of below the label ‘Ultrasound 2.5 Mpa 100 ms’ with central alignment to the figure panels. This has been corrected in both the PDF and HTML versions of the Article.

The original version of the Supplementary Information associated with this Article was missing Supplementary Tables 1 and 2. The HTML has been updated to include a corrected version of the Supplementary Information.

