# Supplementary material for: Sonogenetic control of mammalian cells using exogenous Transient Receptor Potential A1 channels
Source: Nat Commun. 2022 Feb 9;13:600. doi: 10.1038/s41467-022-28205-y (PMC8828769; doi:10.1038/s41467-022-28205-y)
Supplement: Supplementary file 1 — Supplementary Information [file 41467_2022_28205_MOESM1_ESM.pdf]

Sonogenetic control of mammalian cells using exogenous Transient Receptor Potential A1 channels

Marc Duque[1†], Corinne A. Lee-Kubli[1†], Yusuf Tufail[1†], Uri Magaram[1,2], Janki Patel[1], Ahana Chakraborty[1], Jose Mendoza Lopez[1], Eric Edsinger[1], Aditya Vasan[3], Rani Shiao[1], Connor Weiss[1], James Friend[3] and Sreekanth H.Chalasani[1,2*].

[1] Molecular Neurobiology Laboratory, The Salk Institute for Biological Studies, La Jolla, CA 92037.

[2] Neurosciences Graduate Program, University of California San Diego, La Jolla, CA 92093

[3] Medically Advanced Devices Laboratory, Department of Mechanical and Aerospace Engineering, Jacobs School of Engineering and the Department of Surgery, School of Medicine, University of California San Diego, La Jolla, CA 92093.

***Corresponding author. Email: schalasani@salk.edu (S.H.C)**

†These authors contributed equally to this work.

This file includes Supplementary Figures S1-13 and Supplementary Tables S1 and S2.

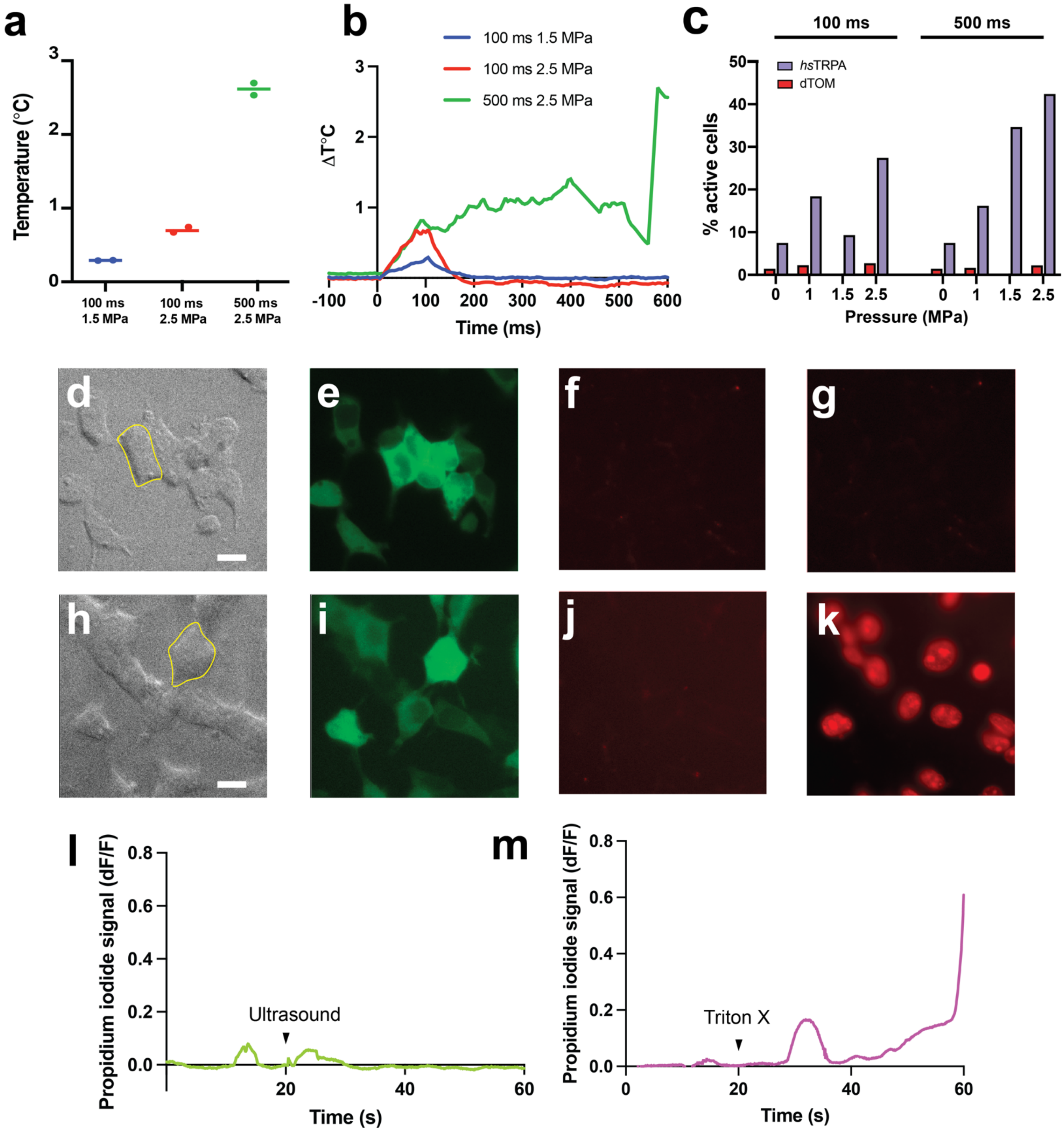
a
Temperature (°C)
3
2
1
0
100 ms 1.5 MPa
100 ms 2.5 MPa
500 ms 2.5 MPa
b
100 ms 1.5 MPa
100 ms 2.5 MPa
500 ms 2.5 MPa
ΔT°C
Time (ms)
-100 0 100 200 300 400 500 600
c
100 ms
500 ms
hsTRPA
dTOM
% active cells
50 40 30 20 10 0
0 1 1.5 2.5 0 1 1.5 2.5
Pressure (MPa)
d
e
f
g
h
i
j
k
l
Propidium iodide signal (dF/F)
0.8 0.6 0.4 0.2 0.0
Ultrasound
20 40 60
Time (s)
m
Propidium iodide signal (dF/F)
0.8 0.6 0.4 0.2 0.0
Triton X
0 20 40 60
Time (s)

Supplementary Figure S1. Safety profile of 7MHz ultrasound stimulation in HEK cells. a, Plot showing maximum temperature increases under different ultrasound stimulation parameters. n = 3 assays/condition. **b**, Time series of ultrasound-evoked temperature changes in the cell culture dish during stimulation. **c**, % active *hs*TRPA1 and dTom transfected cells in response to ultrasound at different pressure and durations. n = 3 coverslips/condition**. d,** Image showing bright field (BF) image for GCaMP6f-HEK cells, and the corresponding GFP channel (**e**) and propidium iodide channel (**f**) before ultrasound stimulation. Multiple trials with ultrasound stimulation at 2.5MPa 100ms had no effect on the intracellular levels of propidium iodide (**g**) n = 3 stims. **h,** Image showing HEK cells used for the positive control, including GFP channel (**i**) and propidium iodide channel before treatment (**j**). Addition of 0.1% Triton-X induced a significant increase of intracellular propidium iodide (**k**). Time course for the propidium iodide signal for an ultrasound stimulated cell highlighted in **d,** shown in **l,** and for a cell treated with propidium iodide highlighted in **h,** shown in **m.** Scale bar, 20 µm.

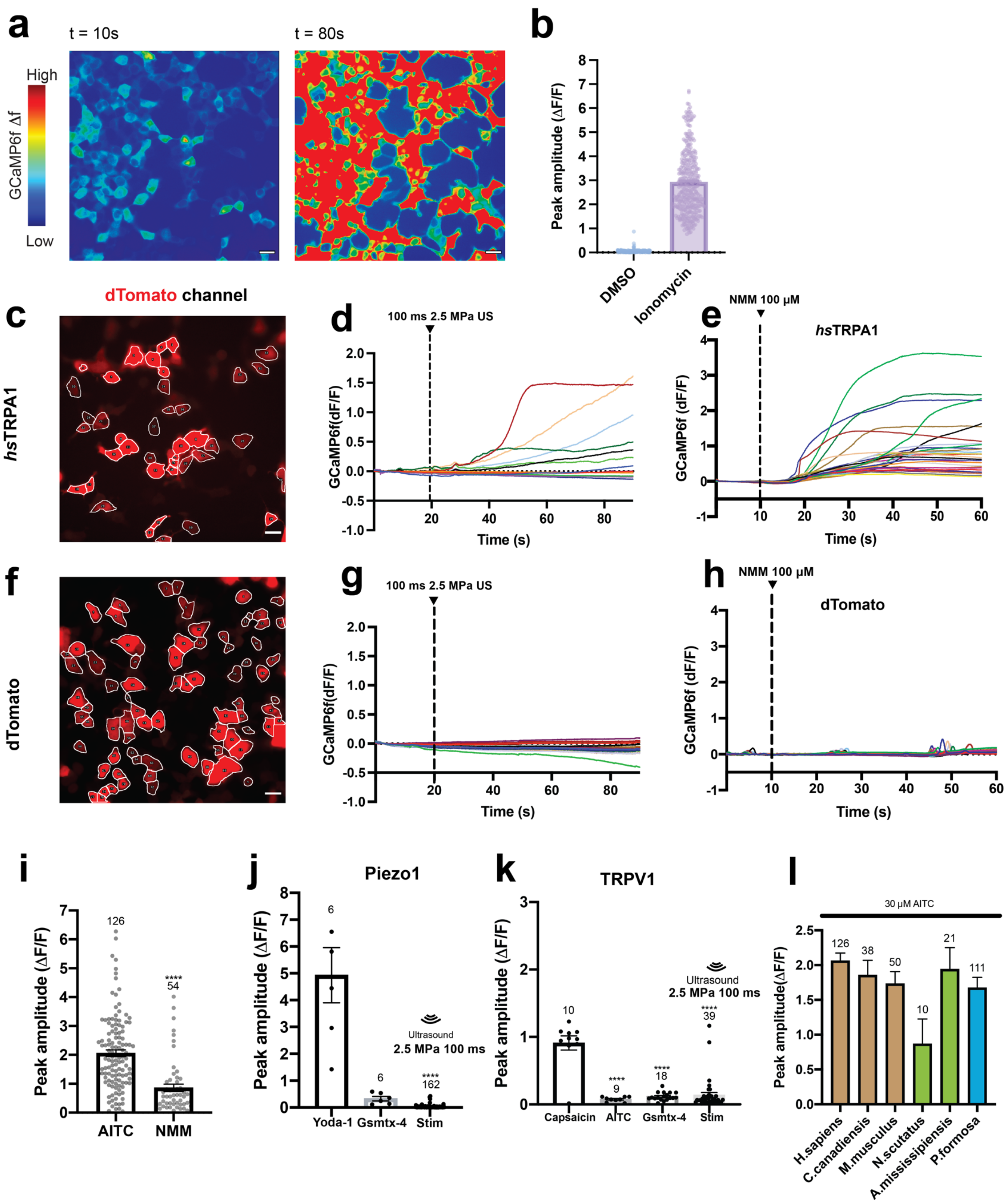

a
t = 10s
t = 80s
High
GCaMP6f Δf
Low
b
Peak amplitude (ΔF/F)
DMSO
Ionomycin
dTomato channel
c
hsTRPA1
d
100 ms 2.5 MPa US
GCaMP6f(dF/F)
Time (s)
e
NMM 100 μM
hsTRPA1
GCaMP6f (dF/F)
Time (s)
f
dTomato
g
100 ms 2.5 MPa US
GCaMP6f(dF/F)
Time (s)
h
NMM 100 μM
dTomato
GCaMP6f (dF/F)
Time (s)
i
Peak amplitude (ΔF/F)
126
****
54
AITC
NMM
j
Piezo1
Peak amplitude (ΔF/F)
6
6
Ultrasound
2.5 MPa 100 ms
****
162
Yoda-1
Gsmtx-4
Stim
k
TRPV1
Peak amplitude (ΔF/F)
10
****
9
****
18
Ultrasound
2.5 MPa 100 ms
****
39
Capsaicin
AITC
Gsmtx-4
Stim
l
30 μM AITC
Peak amplitude(ΔF/F)
126
38
50
10
21
111
H.sapiens
C.canadiensis
M.musculus
N.scutatus
A.mississipiensis
P.formosa

Supplementary Figure S2. Characterization of TRPA1 calcium responses in HEK cells expressing GCaMP6f. **a**, Image showing GCaMP6f ΔF/F before and after treatment with ionomycin (1 μM) in our HEK-GCaMP6f cell line. Scale bar, 25 μm **b**, Individual cell responses as peak GCaMP6f dF/F, to addition of vehicle (DMSO 0.1%) or ionomycin (1uM), showing we can detect intracellular calcium changes in most cells in our field of view. **c,** Image showing example dTom ROIs in HEK cells expressing *hs*TRPA (Scale bar 25 μm) and change in GCaMP fluorescence upon **d,** ultrasound stimulation or **e,** application of NMM in individual cells. **f**, Image showing HEK cells expressing dTom control (Scale bar 25 μm) and change in GCaMP fluorescence upon **g,** ultrasound stimulation in individual cells or **h,** application of NMM in individual cells. **i,** HEK cells expressing *hs*TRPA1 respond to TRPA1 agonists, N-methyl maleimide (NMM, 100 μM). and allyl isothiocyanate (AITC 33 μM). n = 3 coverslips/condition. **j**, HEK cells expressing mouse-Piezo1 respond to yoda-1(10 μM), but not GsMTx-4 or ultrasound. n = 3 coverslips/condition. **k**, HEK cells expressing human-TRPV1 respond to capsaicin (3 μM), but not AITC, GsMTx-4-4 or ultrasound. n = 3 coverslips/condition. Number of cells analyzed is shown on each bar. ****p<0.0001, by Kruskal-Wallis rank test and Dunn's test for multiple comparisons. Scale bar, 20 μm. **l**, Response to AITC in HEK cells expressing TRPA1 from tested species. Data are mean ± SEM (**i, j, k, l,**).

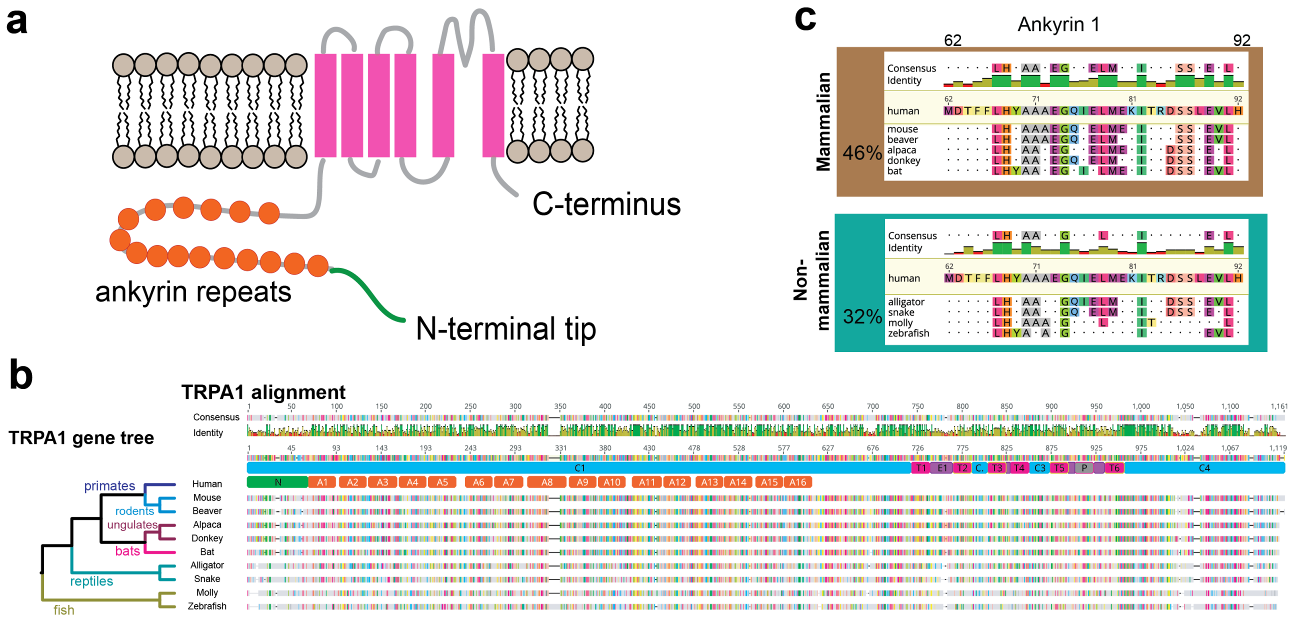

Supplementary Figure S3. TRPA1 sequence alignment across homologs tested for ultrasound sensitivity. a, Schematic of TRPA1 showing the N-terminal region (green), 16 ankyrin repeats (orange) and the 6 transmembrane domains (pink). Mammalian and non-mammalian alignments of TRPA1 homologs tested for ultrasound sensitivity, depicting different domains and %identity compared to hsTRPA1 for the whole protein **b**, and for Ankyrin 1 **c**. % indicates % identity between 65% consensus sequence and *hs*TRPA1.

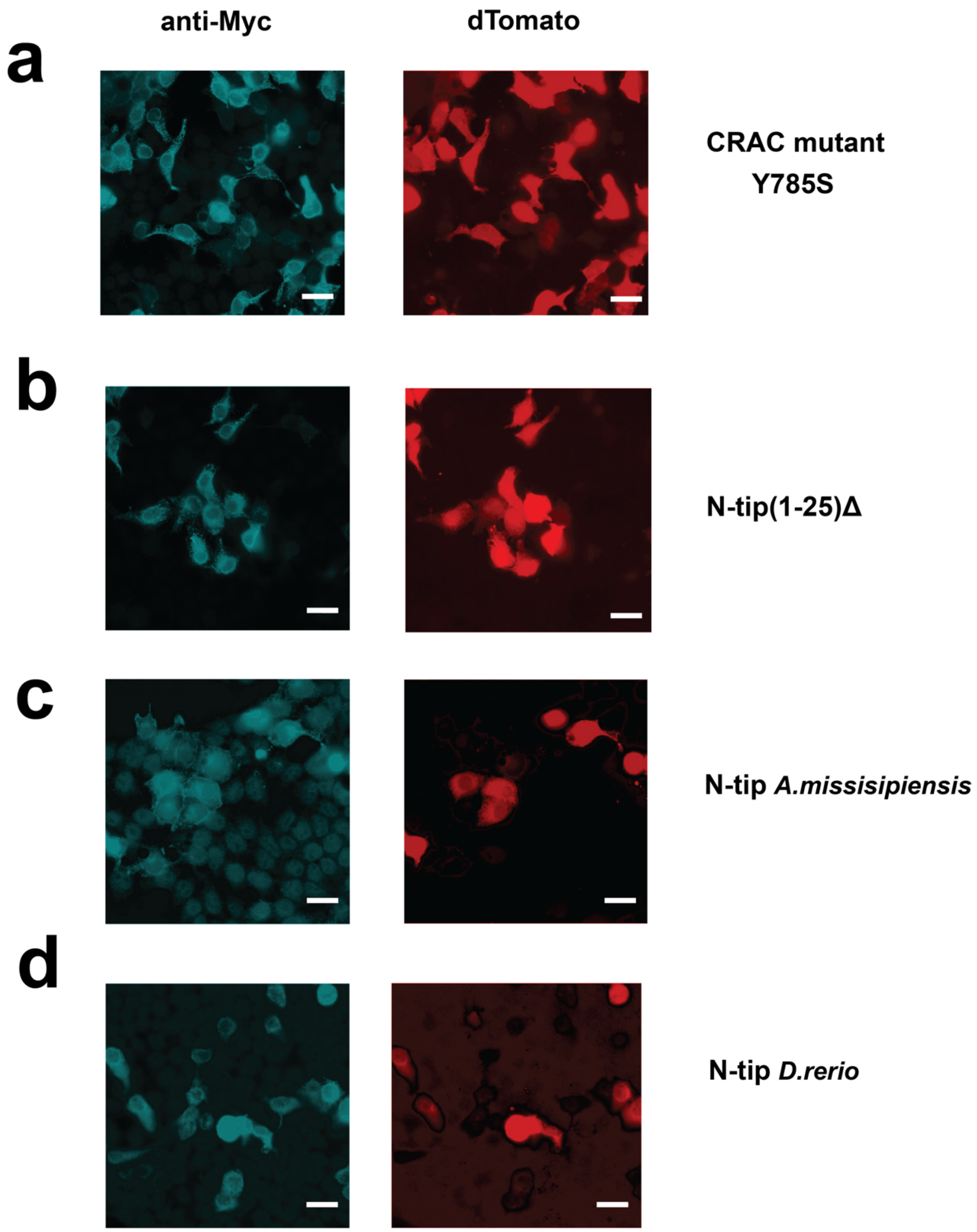

Supplementary Figure S4. Expression of TRPA1 mutants in HEK293T cells. Immunohistochemistry showing expression and correct trafficking of myc-tagged TRPA1 constructs with **a,** CRAC Y785S mutation, **b,** N-terminal tip (1-25) deletion, **c,** *am*TRPA1 N-

terminal tip swapped into *hs*TRPA1 and **d,** *dr*TRPA1 N-terminal tip swapped into *hs*TRPA1. Scale bar 20μm.

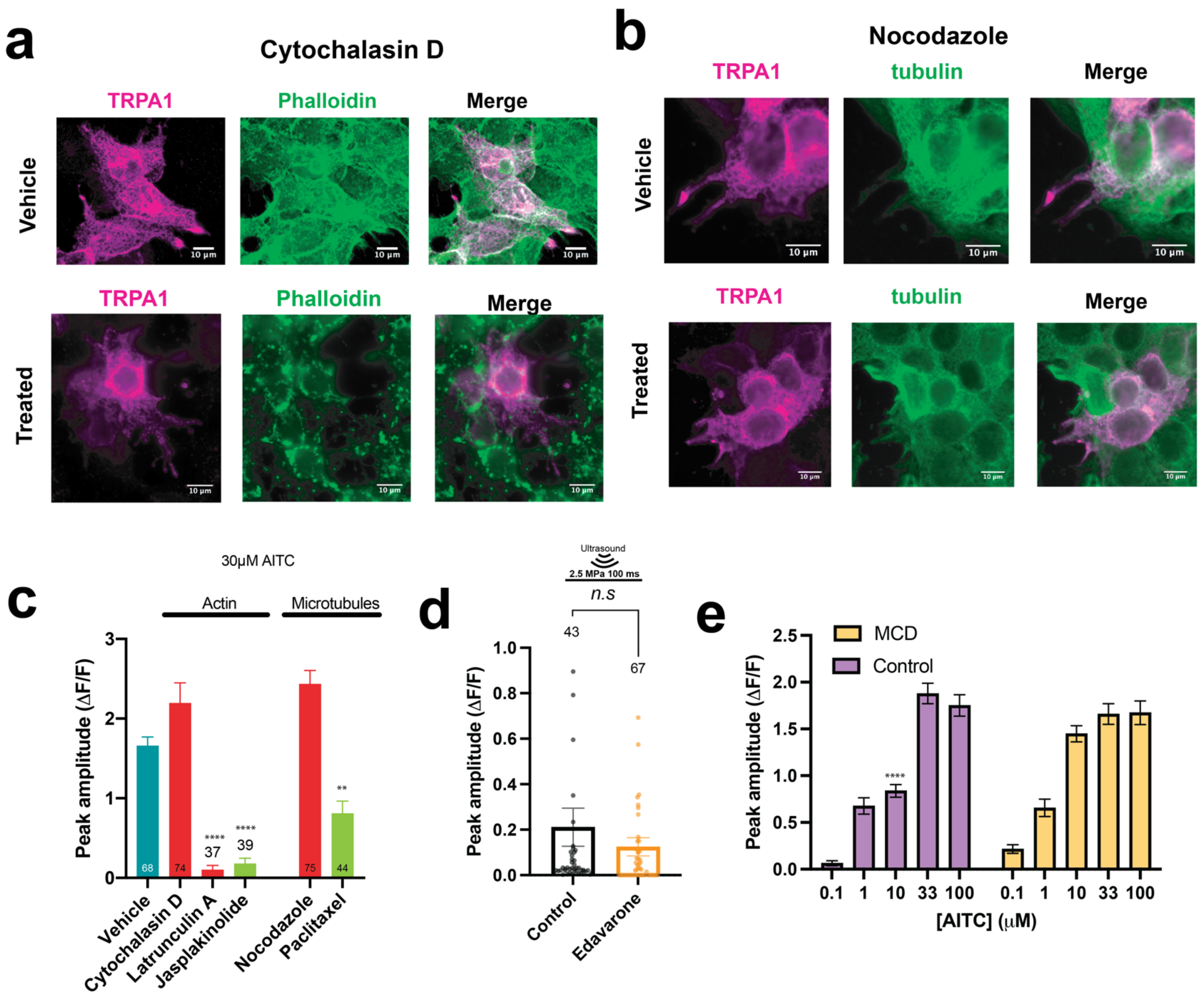

Supplementary Figure S5. Pharmacology to characterize *hs*TRPA1 responses in HEK cells. HEK293 cells expressing *hs*TRPA1 have disrupted **a,** microtubules after treatment with nocodazole, **b**, actin filaments after cytochalasin-D treatment, but not vehicle controls. Microtubules are labeled using anti-alpha tubulin, while actin filaments are assessed by phalloidin staining. **c,** Treating HEK293 cells expressing *hs*TRPA1 with cytochalasin D or nocodazole has no significant effect on AITC responses compared to vehicle controls. In contrast, HEK293-*hs*TRPA responses to AITC were reduced after treatment latrunculin A and jasplakinolide and paclitaxel, presumably due to poor cell health. n = 3 coverslips/condition. Numbers of cells analyzed are shown in each bar. ** $p < 0.01$, **** $p < 0.0001$ Kruskal-Wallis rank test and Dunn's

test for multiple comparison **d,** HEK293 expressing hsTRPA1 respond to ultrasound independent of treatment of a reactive oxygen blocker (Edavarone 10µM). N= 3 coverslips/condition. Numbers of cells analyzed are shown on each bar. n.s, not significant $p>0.05$ by Mann-Whitney test**. e,** Responses as mean peak ΔF/F GCaMP6f amplitude in control HEK-*hs*TRPA1 cells (control) and cells treated with 5mM methyl-β-cyclodextrin (MCD) for 1h, to different allyl isothiocyanate (AITC) concentrations. Vehicle is DMSO (0.1%). N= 3 coverslips/condition. All conditions are not significant ($p>0.5$) between MCD vs. control, except for 10µM, $p<0.0001$ by two-way ANOVA and Šídák's multiple comparisons test. Data are mean ± SEM (**c, d, e**). p-value = 0.0511 (**d**).

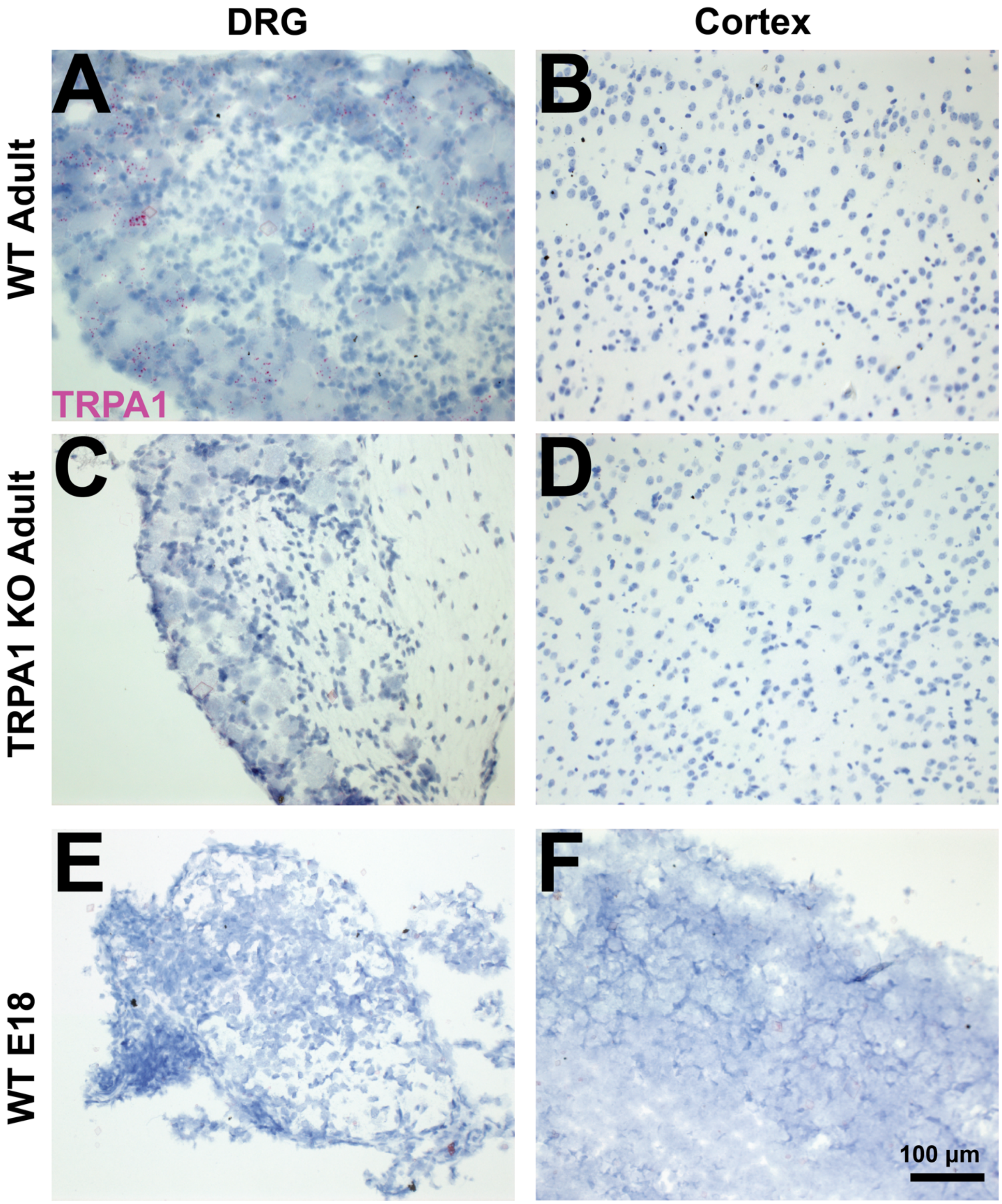

Supplementary Figure S6. TRPA1 RNA is not detected in the E18 or adult mouse cortex. Results from a Base Scope *in situ* hybridization experiment in adult DRG and cortex taken from **(a, b)** wild-type (WT) C57Bl6/J mouse or **(c, d)** TRPA1 -/- mice as well as E18 **(e)** DRG and **(f)** cortex taken from a WT C57Bl6 embryo. Positive signal is detected as magenta puncta within cell bodies and was only detected in the adult WT DRG, as expected.

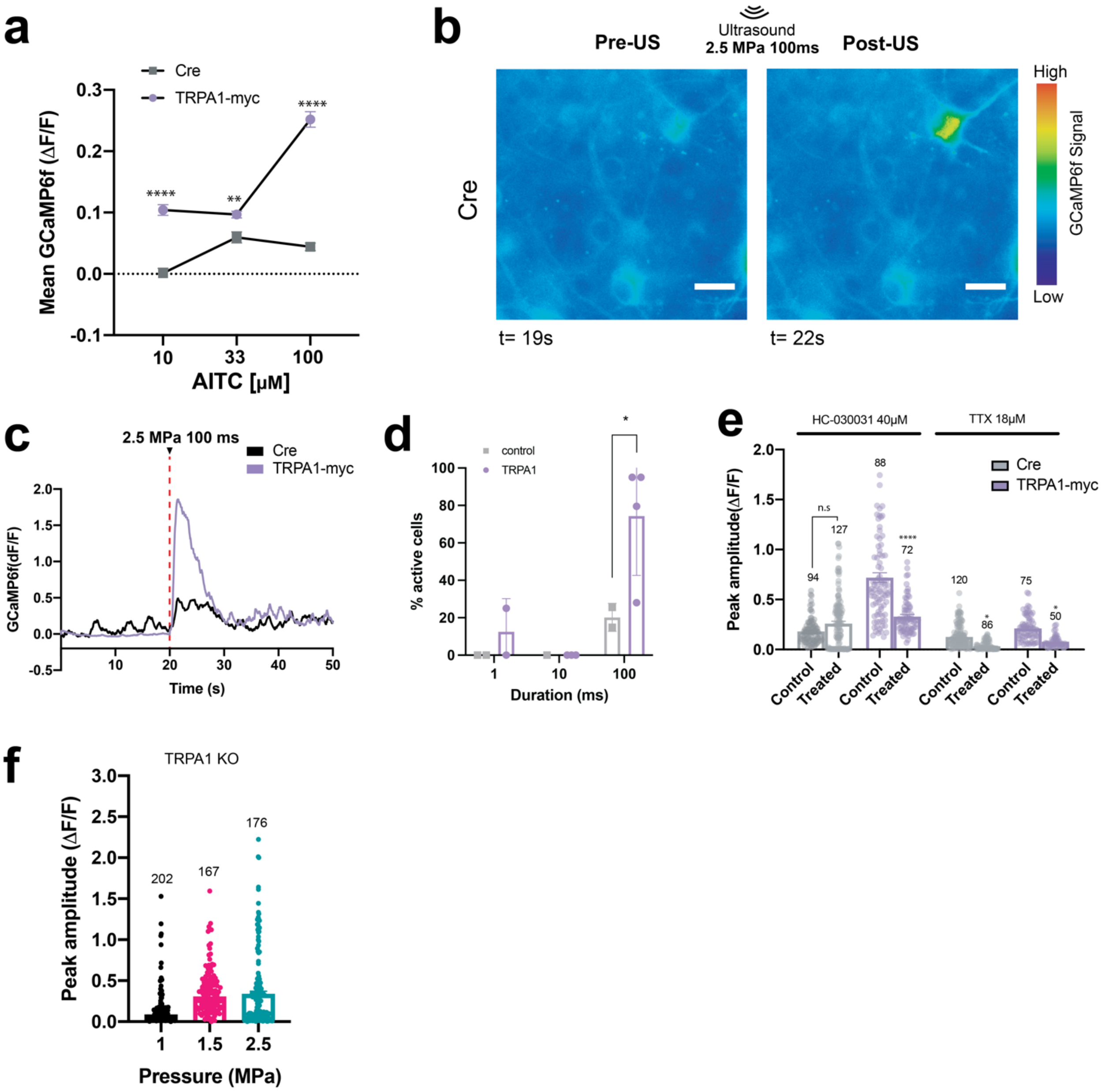
a
Cre
TRPA1-myc
Mean GCaMP6f (ΔF/F)
0.3
0.2
0.1
0.0
-0.1
****
**
****
10
33
100
AITC [μM]
b
Ultrasound
2.5 MPa 100ms
Pre-US
Post-US
Cre
t= 19s
t= 22s
High
Low
GCaMP6f Signal
c
2.5 MPa 100 ms
Cre
TRPA1-myc
GCaMP6f(dF/F)
Time (s)
d
control
TRPA1
% active cells
Duration (ms)
*
e
HC-030031 40μM
TTX 18μM
Cre
TRPA1-myc
Peak amplitude(ΔF/F)
n.s
94
127
88
72
****
120
86
75
50
Control
Treated
f
TRPA1 KO
Peak amplitude (ΔF/F)
202
167
176
Pressure (MPa)

Supplementary Figure S7. Ultrasound-evoked responses in control primary neurons are independent of TRPA1. (**a**) Dose response curve of *hs*TRPA1-, and Cre-control expressing neurons to AITC. N = 3 coverslips/condition. ** $p < 0.01$, **** $p < 0.0001$ compared to Cre controls with two way ANOVA followed by Sidak's multiple comparisons test. (**b**) Image showing GCaMP fluorescence in primary neurons infected with control Cre virus before and after ultrasound stimulation. Scale bar 20 μm. (**c**) Representative traces showing magnitude of ultrasound-induced responses in representative control (Cre) or hsTRPA1-expressing neurons. (**d**) Responses as % active cells in both control and TRPA1 expressing primary neurons to one pulse of 2.5MPa 7MHz ultrasound stimulation at 1, 10 and 100ms duration. N = 3 coverslips/condition. * $p < 0.05$ Mann-Whitney Test. (**e**) GCaMP6f ΔF/F peak amplitude for both control and *hs*TRPA1 expressing primary neurons in response to 2.5MPa 100ms 7MHz ultrasound pulse under different treatments. Cells were treated with either TRPA1 antagonist (HC-030031) or the sodium channel blocker tetradotoxin (TTX) for 30 mins and then imaged. N = 3 coverslips/condition. * $p < 0.05$, **** $p < 0.0001$, n.s not significant $p > 0.05$ compared to controls with two way ANOVA followed by Sidak's multiple comparisons test. (**f**) Primary neurons from TRPA1 knockout mice responded to ultrasound. N = 3 coverslips/condition. Number of cells analyzed is shown in each bar. .* p<0.05 by two-way ANOVA and Šídák's multiple comparisons test (**d**) and n.s p>0.5,* p<0.05, **** p<0.001 by one-way ANOVA. Data are mean ± SEM (**a,d,e,f**). p-value = 0.0477 (**d**).

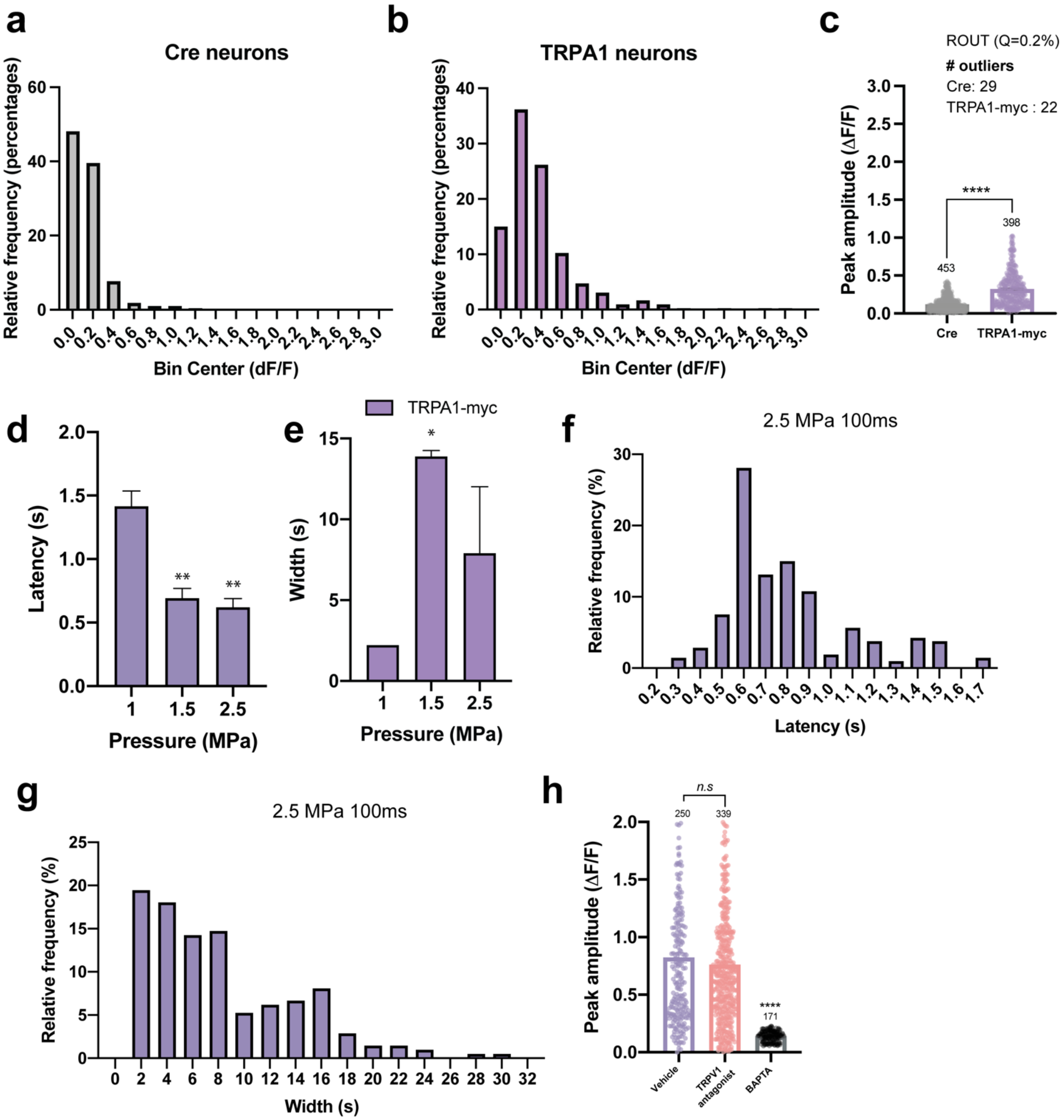
a
Cre neurons
Relative frequency (percentages)
Bin Center (dF/F)
b
TRPA1 neurons
Relative frequency (percentages)
Bin Center (dF/F)
c
ROUT (Q=0.2%)
outliers
Cre: 29
TRPA1-myc : 22
Peak amplitude (ΔF/F)
****
453
398
Cre
TRPA1-myc
TRPA1-myc
d
Latency (s)
Pressure (MPa)
e
Width (s)
Pressure (MPa)
f
2.5 MPa 100ms
Relative frequency (%)
Latency (s)
g
2.5 MPa 100ms
Relative frequency (%)
Width (s)
h
n.s
250
339
****
171
Peak amplitude (ΔF/F)
Vehicle
TRPV1 antagonist
BAPTA

Supplementary Figure S8. Characterizing ultrasound responses in *hs*TRPA1 expressing primary neurons. (**a**) Distribution of ultrasound responses to 100ms 2.5MPa in control and **b**, *hs*TRPA1-myc primary neurons. **c**, removing outliers reduces the maximum value observed for *hs*TRPA1-myc infected neurons but we still observe a statistically significant difference between controls and *hs*TRPA1 (pv<0.001) confirming the robustness of the effect. Plot showing **d**, time to 60% of peak response (latency) and **e**, time between 63% rise and 63% decay (response width) after ultrasound stimulation at 100 msecs and different peak negative pressures in *hs*TRPA1 expressing primary neurons. Plots showing distribution of **f,** latency) and **g,** response width after ultrasound stimulation in *hs*TRPA1 expressing neurons. (**h**) Plot showing GCaMP6f peak amplitude in *hs*TRPA1 expressing neurons after ultrasound stimulation and treatment with either TRPV1 antagonist (A784168, 2 µM), Calcium chelator (BAPTA, 30 µM) or vehicle (DMSO). n = 3 coverslips/condition. Numbers of cells analyzed is shown in each bar. * p<0.05, ** p<0.01 by one-way ANOVA, (**h**) ****p<0.0001, n.s, not significant p>0.05 by Kruskal-Wallis rank test and Dunn's test for multiple comparisons. Data are mean ± SEM (**c, d, e**).

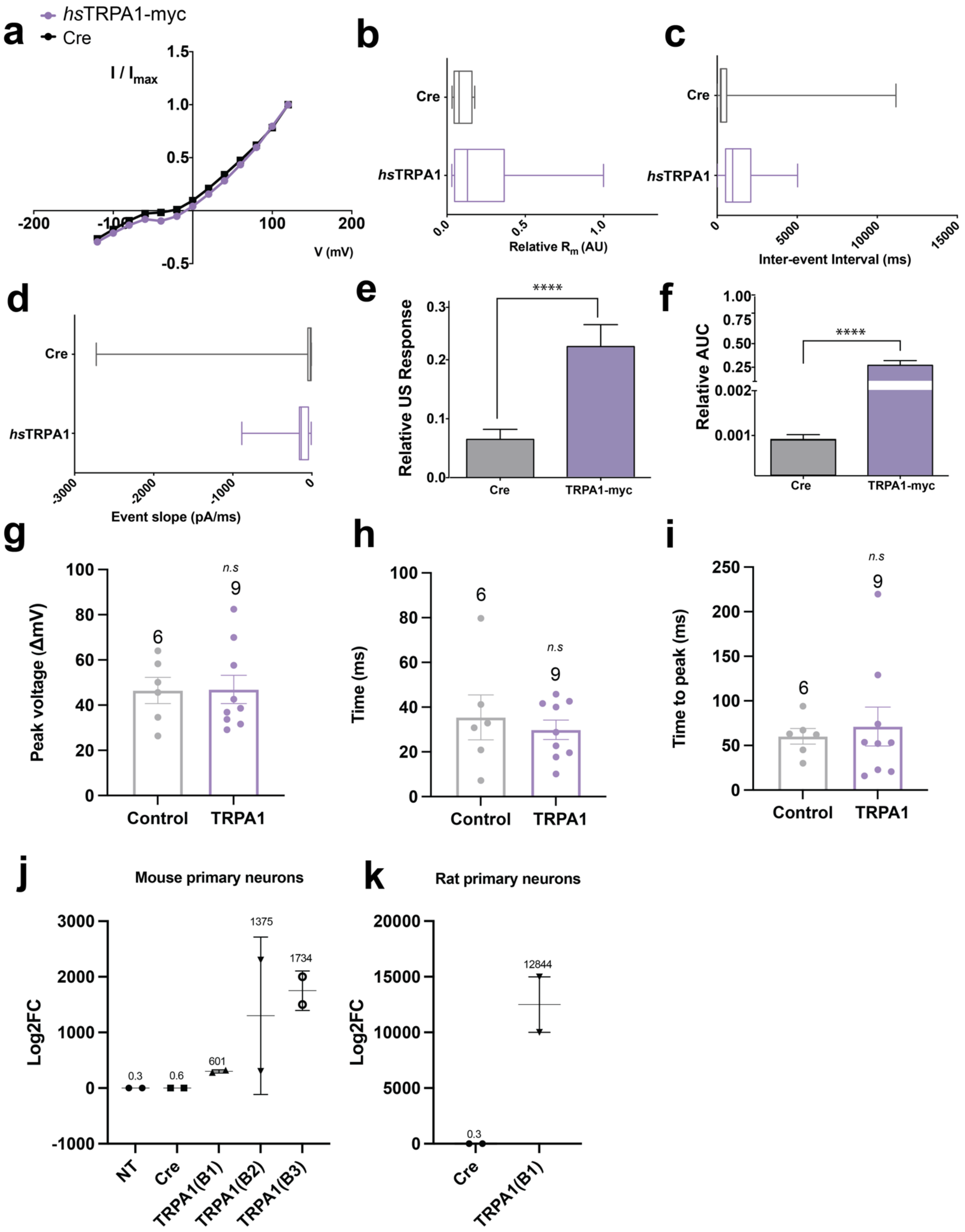

a
hsTRPA1-myc
Cre
I / Imax
V (mV)
b
Cre
hsTRPA1
Relative Rm (AU)
c
Cre
hsTRPA1
Inter-event Interval (ms)
d
Cre
hsTRPA1
Event slope (pA/ms)
e
Relative US Response
****
Cre
TRPA1-myc
f
Relative AUC
****
Cre
TRPA1-myc
g
Peak voltage (ΔmV)
n.s
Control
TRPA1
h
Time (ms)
n.s
Control
TRPA1
i
Time to peak (ms)
n.s
Control
TRPA1
j
Mouse primary neurons
Log2FC
0.3
0.6
601
1375
1734
NT
Cre
TRPA1(B1)
TRPA1(B2)
TRPA1(B3)
k
Rat primary neurons
Log2FC
0.3
12844
Cre
TRPA1(B1)

Supplementary Figure S9. Electrophysiological properties of rat primary neurons. Functional and membrane properties are similar between *hs*TRPA1 and Cre-control infected neurons. Current-Voltage (IV) plots **a,** for AAV9-*hs*TRPA1 vs AAV9-Cre control primary neurons elicit similar responses. Membrane resistance can be used as a proxy for patch and recording quality (p=0.36 Unpaired t-test with Welch's correction) **b**, similar Rm was observed for both groups. Other response characteristics including inter-event interval (p=0.174 Unpaired t-test with Welch's correction) **c,** and response slopes (p=0.38 Unpaired t-test with Welch's correction). **d,** were not significantly altered between *hs*TRPA1 and Cre-control infected neurons. **e,** Relative response to ultrasound was significantly increased in *hs*TRPA1-expressing neurons, as was **f,** AUC of the response. N=5 cells/group. Ultrasound induced action potentials show similar metrics both in control and *hs*TRPA1 expressing neurons, including the peak voltage (**g**), latency relative to ultrasound stimulus (**h**) and time to peak (**i**). Expression assessed by qPCR of *hs*TRPA1 transcript is comparable in mouse (**j**) and rat (**k**) primary neurons. Reads for non-transfected (NT), AAV-Cre only control (Cre) and different batches of AAV-TRPA1 (B1,B2,B3) are shown. N = 2 coverslips/condition. Data are mean ± SEM (**e, f, g, h, i**). Box plots (**b**, and **c**) show min, Q1, median, Q3 and max.

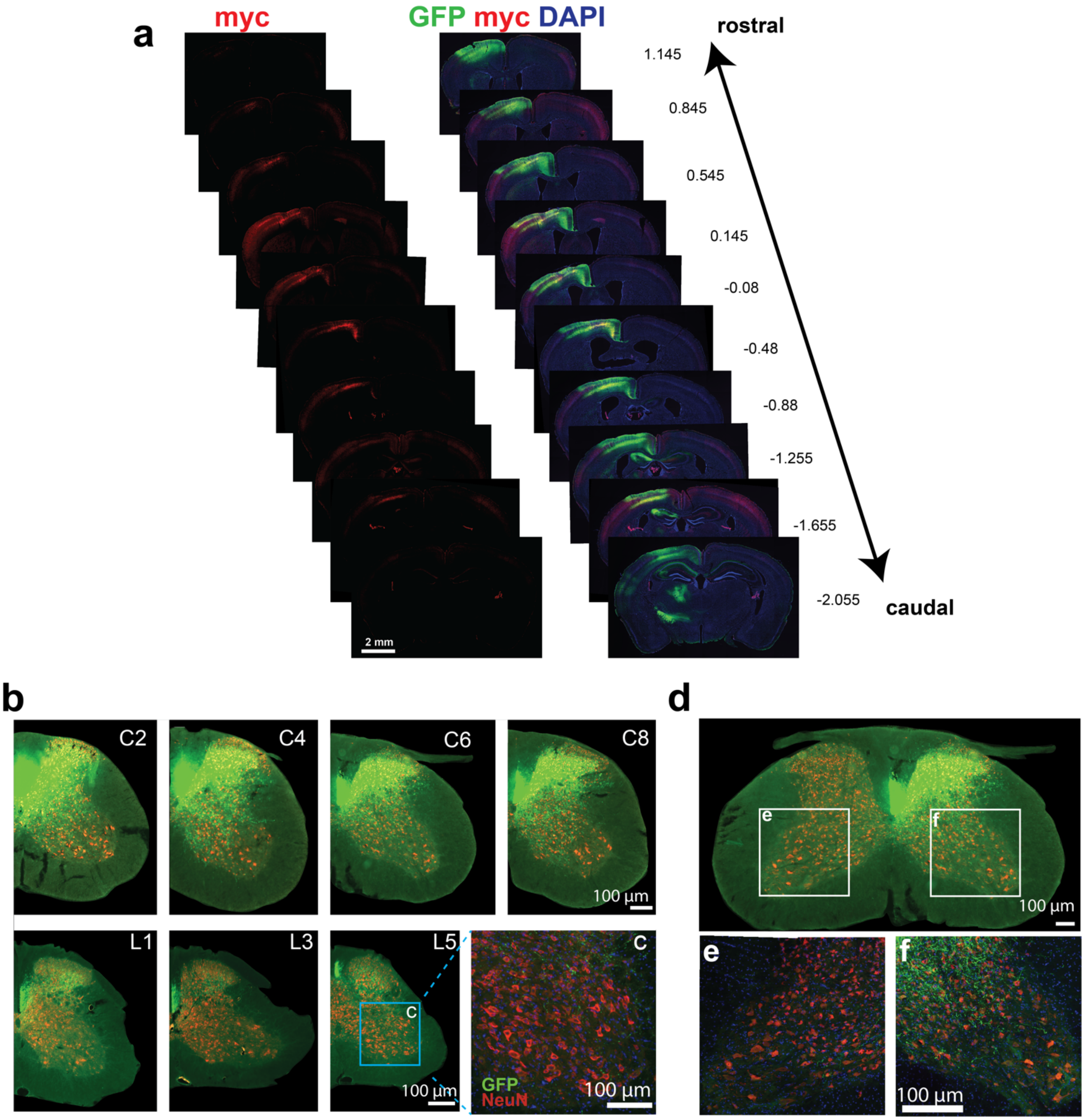

Supplementary Figure S10. myc-*hs*TRPA1 expresses in forelimb and hindlimb motor cortex, innervating lumbar and cervical spinal cord. (**a**) Brain sections taken every ~350 µM were immunolabeled for myc, GFP and DAPI to evaluate the rostro caudal extent of viral expression. Approximate AP coordinates are taken from (Allen Brain Atlas(*31*)). **b**, Spinal cord sections taken every ~875 µM were immunolabeled for GFP and NeuN to evaluate the projection pattern of Npr3-Cre neurons that took up injected virus. Images are from a mouse that received co-injection of 4E13 myc-*hs*TRPA1 and 1 E12 GFP. Images were collected at 10x. **c,** A 20x confocal image of the inset from L5 showing GFP+ axons innervating the ventral horn. **d,** C6 spinal cord from the same mouse showing GFP+ axons in the **e**, ipsilateral and **f,** contralateral ventral horns.

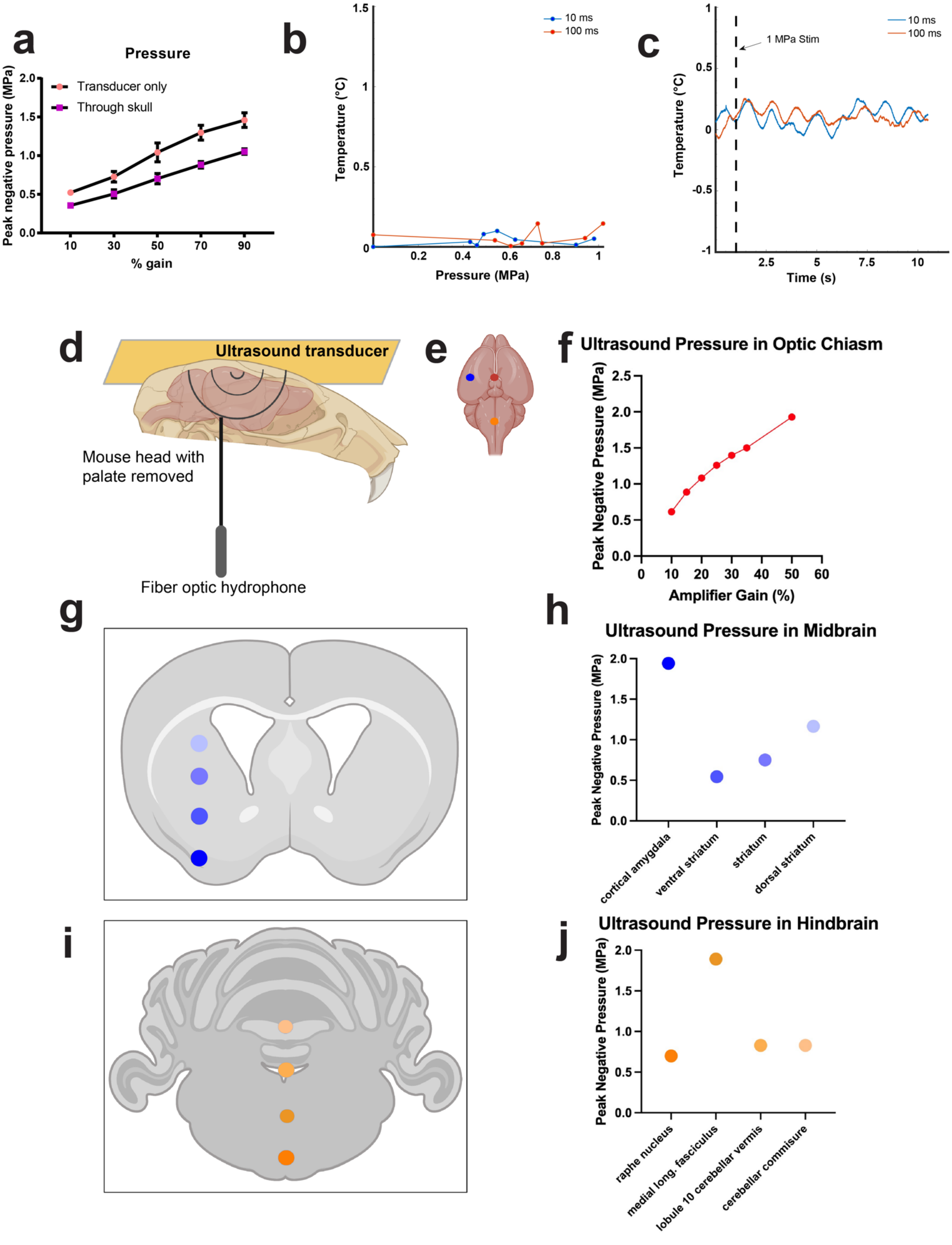

a
Pressure
Peak negative pressure (MPa)
Transducer only
Through skull
% gain
b
10 ms
100 ms
Temperature (°C)
Pressure (MPa)
c
1 MPa Stim
10 ms
100 ms
Temperature (°C)
Time (s)
d
Ultrasound transducer
Mouse head with
palate removed
Fiber optic hydrophone
e
f
Ultrasound Pressure in Optic Chiasm
Peak Negative Pressure (MPa)
Amplifier Gain (%)
g
h
Ultrasound Pressure in Midbrain
Peak Negative Pressure (MPa)
cortical amygdala
ventral striatum
striatum
dorsal striatum
i
j
Ultrasound Pressure in Hindbrain
Peak Negative Pressure (MPa)
raphe nucleus
medial long. fasciculus
lobule 10 cerebellar vermis
cerebellar commisure

Supplementary Figure S11. Pressure-temperature profile of ultrasound delivery *in vivo*. a, Pressure profile of the ultrasound transducer used for *in vivo* experiments. Peak negative pressure was measured at a consistent location relative to the face of the transducer either through ultrasound gel, or in the cortex while the ultrasound transducer was coupled to the skull with ultrasound gel. Transducer pressure output increased as a function of changing the % gain on the amplifier. **b,** Peak temperature change measured 1mm from the face of the transducer or in the cortex in response to 10 and 100ms ultrasound stimulation at increasing pressures (reported pressures are those measured within the cortex). **c**, Representative temperature traces recorded within the cortex in response to stimulation at 1 MPa peak negative pressure at 10 or 100 ms stimulus durations. **d**, Schematic of hydrophone recordings in ex vivo mouse brain, with skull intact and palate removed. **e**, Red dot indicates hydrophone location at optic chiasm, ventral-most part of the brain. Orange and blue dots indicate subsequent measurements at constant power and variable depth. **f**, Transducer can deliver >1.5MPa to deepest portions of the brain for sonogenetic applications. **g**, Representative midbrain coronal section, with blue dots representing hydrophone measurement locations. **h**, Ultrasound pressure delivered to midbrain (30% amplifier gain), increased power can compensate for mid-range pressures. **i**, Representative hindbrain coronal section, with orange dots representing hydrophone measurement locations. **j**, Ultrasound pressure delivered to hindbrain (30% amplifier gain), increased power can compensate for mid-range pressures. Data are mean ± SEM (**a**).

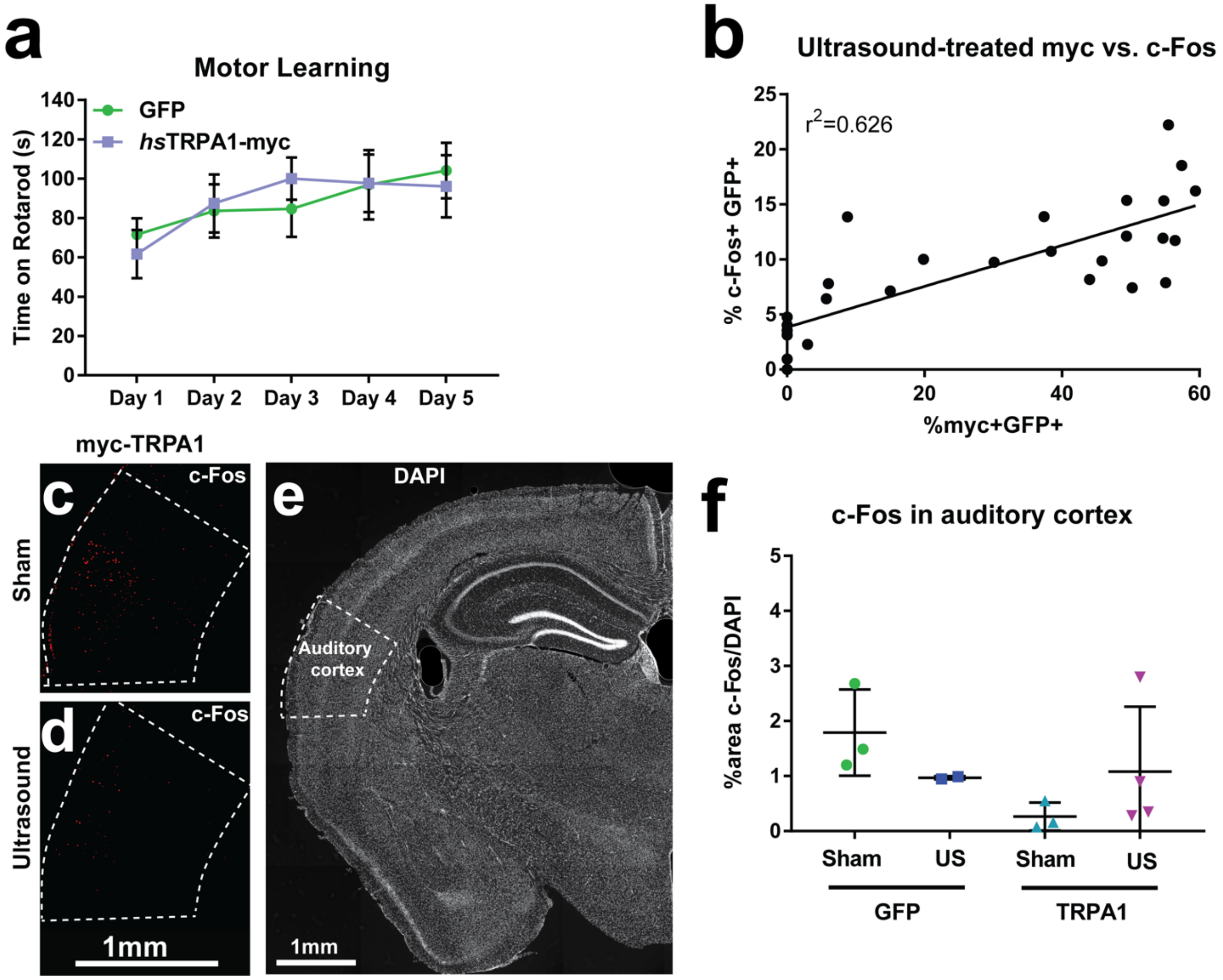

Supplementary Figure S12. Supplemental data from the *in vivo* experiments. a, Rotarod performance in mice injected with 1E12 AAV9-hsyn-DIO-GFP or 1E14 AAV9-hsyn-DIO-myc:*hs*TRPA1. N=6-7 per group. No significant differences between groups by two-way ANOVA and Sidak's multiple comparisons test. Both groups showed significant improvement in rotarod performance over the 5 days. P<0.0003 Day 5 compared to Day 1 by two-way ANOVA and Tukey's multiple comparisons test. **b,** Correlation between %c-fos+/GFP+ neurons to %myc+/GFP+ neurons across adjacent individual sections from mice that received ultrasound treatment. R^2=0.626. P=0<0.001. Images of c-fos in the auditory cortex from myc-*hs*TRPA1-expressing mice that received **(c)** sham stimulation or **(d)** 1hr of 100msec 1.05MPa stimulation delivered every 10 secs. **e,** Anatomical localization of auditory cortex in DAPI-labelled tissue. **f,** Quantification of % area of auditory cortex containing c-fos+ signal normalized to % area of the DAPI signal. No significant differences were detected across groups by One-way ANOVA. Data are mean ± SEM (**a,f**).

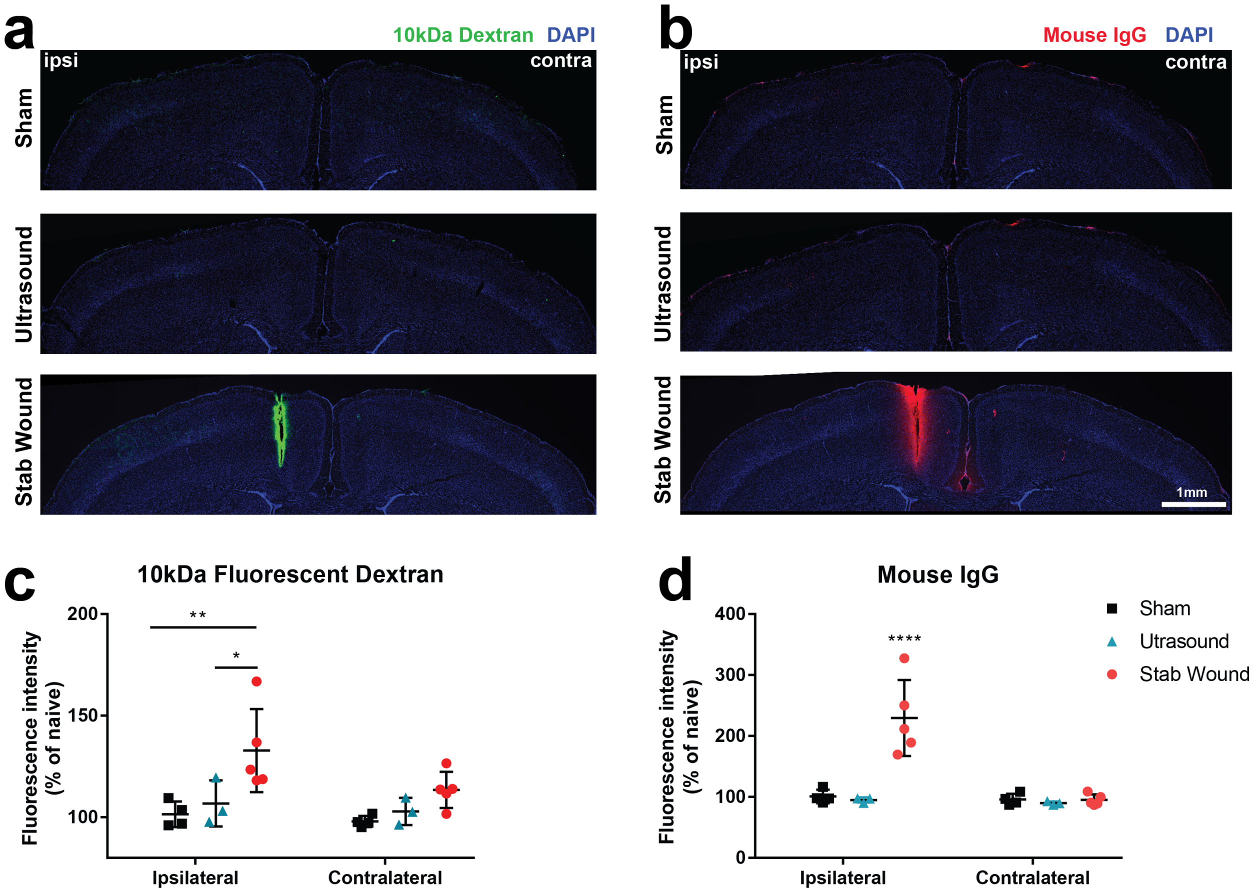

Supplementary Figure S13. The blood brain barrier is not disrupted by 1 hr of intermittent 100ms ultrasound delivered at 1.0 MPa. Representative images of **a,** cortical fluorescent dextran and **b,** mouse IgG immunolabeling across conditions. **c**, Quantification of 10 kDa fluorescent dextran in each cortical hemisphere from mice that were treated with either ultrasound (100 ms, 1.0 MPa every 10 s) or sham stimulation for 1 hour or that had received a cortical stab wound condition, normalized to the cortical fluorescence of uninjected naïve mice. **d**, Quantification of mouse IgG in in each cortical hemisphere from mice that were treated with either ultrasound (100 ms, 1.0 MPa every 10 s) or sham stimulation for 1 hour or that had received a cortical stab wound condition, normalized to the cortical fluorescence of uninjected naïve mice. *$p<0.05$, **$p<0.01$, ****$p<0.0001$ by two-way ANOVA followed by Sidak's multiple comparison's test. N=3-5/group. Data are mean ± SEM (**c,d**).

Supplementary Table S1. Table showing a library of 191 clones from various protein families including DEG/ENaC, K2P, TRP, ASIC, Piezo, MscS, MscL and Prestin from multiple different species.

Protein family	# Clones	# Species	Kingdom(s)
ASIC	12	6	Animals
KCNK	2	1	Animals (human)
MSCL	13	4	Bacteria
MSL	4	1	Plants
PIEZO	8	2	Animals (human,mouse)
PKD1	1	1	Animals
Prestin	7	5	Animals
SCNN	7	6	Animals
TRPA1	10	10	Animals
TRPC	4	3	Animals
TRPM	9	4	Animals
TRPN	8	5	Animals (zebrafish, cnidaria, drosophila)
TRPV	8	5	Animals (human, brown bat, killer whale)
Other	99	21	Animals, Plants, Bacteria
**Total**	**191**		

Supplementary Table S2. Table showing percent identity across all TRPA1 domains based on pair-wise alignment of consensus sequence for tested chordate, mammalian, and non-mammalian clades compared to human. Percent identity marked in red indicates regions that are particularly conserved or divergent between mammals and non-mammalian chordates. Threshold for consensus is bases matching to human reference in 65% of sequences in multiple sequence alignments of each clade.

ID	Domain	Uniprot (start-stop)	Percent Identity Human x Chordate Consensus 65%	Percent Identity Human x Mammal Consensus 65%	Percent Identity Human x Non-Mammal Consensus 65%
TRPA1	Protein	1	65	79	46
N	N-terminus	1-61	25	58	13
A1	Ankyrin 1	62-92	44	46	32
A2	Ankyrin 2	97-126	73	87	58
A3	Ankyrin 3	130-160	50	67	36
A4	Ankyrin 4	164-193	61	71	45
A5	Ankyrin 5	197-226	66	69	52
A6	Ankyrin 6	238-267	69	72	53
A7	Ankyrin 7	271-301	78	84	60
A8	Ankyrin 8	308-337	74	80	66
A9	Ankyrin 9	341-370	82	97	55
A10	Ankyrin 10	374-403	71	90	49
A11	Ankyrin 11	412-441	82	93	52
A12	Ankyrin 12	445-474	92	97	74
A13	Ankyrin 13	481-510	94	100	71
A14	Ankyrin 14	513-542	84	93	57
A15	Ankyrin 15	547-576	74	88	61
A16	Ankyrin 16	579-609	58	80	44
C1	Cytoplasmic Domain 1	1	NA	NA	NA
T1	Transmembrane Domain 1	720-740	75	89	47
E1	Extracellular Domain 1	741-764	32	84	8
T2	Transmembrane Domain 2	765-785	59	86	26
C2	Cytoplasmic Domain 2	786-803	73	78	47
T3	Transmembrane Domain 3	804-824	68	73	34
E2	Extracellular Domain 2	825-829	62	62	5
T4	Transmembrane Domain 4	830-850	73	81	54
C3	Cytoplasmic Domain 3	851-873	67	96	53
T5	Transmembrane Domain 5	874-894	68	82	50
E3	Extracellular Domain 3	895-901	73	72	32
P	Pore Region	902-922	77	81	54
E4	Extracellular Domain 4	923-934	68	75	36
T6	Transmembrane Domain 6	935-956	61	78	38
C4	Cytoplasmic Domain 4	957-1119	59	74	45
