# Supplementary material for: Sonogenetic control of mammalian cells using exogenous Transient Receptor Potential A1 channels
Source: Nat Commun. 2022 Feb 9;13:600. doi: 10.1038/s41467-022-28205-y (PMC8828769; doi:10.1038/s41467-022-28205-y)
Supplement: Supplementary file 2 — Reporting Summary [file 41467_2022_28205_MOESM2_ESM.pdf]

nature portfolio

Corresponding author(s): Sreekanth Chalasani

Last updated by author(s): Jan 8, 2022

Reporting Summary

Nature Portfolio wishes to improve the reproducibility of the work that we publish. This form provides structure for consistency and transparency in reporting. For further information on Nature Portfolio policies, see our Editorial Policies and the Editorial Policy Checklist.

Statistics

For all statistical analyses, confirm that the following items are present in the figure legend, table legend, main text, or Methods section.

n/a	Confirmed	
☐	☒	The exact sample size (*n*) for each experimental group/condition, given as a discrete number and unit of measurement
☐	☒	A statement on whether measurements were taken from distinct samples or whether the same sample was measured repeatedly
☐	☒	The statistical test(s) used AND whether they are one- or two-sided<br>*Only common tests should be described solely by name; describe more complex techniques in the Methods section.*
☐	☒	A description of all covariates tested
☐	☒	A description of any assumptions or corrections, such as tests of normality and adjustment for multiple comparisons
☐	☒	A full description of the statistical parameters including central tendency (e.g. means) or other basic estimates (e.g. regression coefficient) AND variation (e.g. standard deviation) or associated estimates of uncertainty (e.g. confidence intervals)
☐	☒	For null hypothesis testing, the test statistic (e.g. *F*, *t*, *r*) with confidence intervals, effect sizes, degrees of freedom and *P* value noted<br>*Give P values as exact values whenever suitable.*
☒	☐	For Bayesian analysis, information on the choice of priors and Markov chain Monte Carlo settings
☒	☐	For hierarchical and complex designs, identification of the appropriate level for tests and full reporting of outcomes
☒	☐	Estimates of effect sizes (e.g. Cohen's *d*, Pearson's *r*), indicating how they were calculated

Our web collection on statistics for biologists contains articles on many of the points above.

Software and code

Policy information about availability of computer code

Data collection	For calcium imaging HC Image Live (last version available) was used<br>For EMG recordings, LabChart8 (AD Instruments) was used<br>For patch-clamp electrophysiology experiments we used pClamp11 from Molecular Devices
Data analysis	Calcium imaging videos were analyzed using Fiji (https://fiji.sc) and then processed using a custom analysis pipeline which can be accessed at https://github.com/shreklab/Duque-Lee-Kubli-Tufail2020<br>EMG data were analyzed with LabChart8 (AD Instruments).<br>Patch-clamp data was analyzed using pClamp11 software<br>Sequence data was analyzed using Geneious Prime (version 2020.1.2).<br>Sequences were aligned using Geneious Prime MAFFT (ver 7.450).<br>Phylogenetic trees were generated using Geneious Prime RAxML (ver 8.2.11).<br>Maximum likelihood tree was assessed and annotated in FigTree (ver 1.4.4).<br>Motifs were annotated using Geneious Prime EMBOSS 6.5.7 fuzzpro tool

For manuscripts utilizing custom algorithms or software that are central to the research but not yet described in published literature, software must be made available to editors and reviewers. We strongly encourage code deposition in a community repository (e.g. GitHub). See the Nature Portfolio guidelines for submitting code & software for further information.

Data

Policy information about availability of data

All manuscripts must include a data availability statement. This statement should provide the following information, where applicable:

- Accession codes, unique identifiers, or web links for publicly available datasets
- A description of any restrictions on data availability
- For clinical datasets or third party data, please ensure that the statement adheres to our policy

Source data is included in this submission. Publically available sequence data was also used. Indicated in the Data availability section.

Field-specific reporting

Please select the one below that is the best fit for your research. If you are not sure, read the appropriate sections before making your selection.

[x] Life sciences   [ ] Behavioural & social sciences   [ ] Ecological, evolutionary & environmental sciences

For a reference copy of the document with all sections, see nature.com/documents/nr-reporting-summary-flat.pdf

Life sciences study design

All studies must disclose on these points even when the disclosure is negative.

Sample size	For the in vitro experiments, a minimum number of 3 coverslips (50-100 analyzed cells/coverslips) per condition was chosen, since it is the usual number of samples used in calcium imaging methods, and sufficiently large to get statistics on the ultrasound effect. These sample sizes are similar to what has been used in Oh et al (ref#13) and Yoo etl al (ref #16)<br>For patch-clamp electrophysiology experiments, at least 5 cells were recorded and analyzed per condition.<br>For in vivo experiments, n=6 per TRPA1 or GFP injection condition was planned on the basis of effect sizes observed in preliminary studies. However, for EMG studies, n=1 mouse/group succumbed under anesthesia, so final group sizes for the EMG experiment were n=5. For rotarod n=6-7 TRPA1 or GFP-injected mice were prepared on the basis of effect sizes in preliminary studies. For c-fos, n=3-4 mice per virus/ ultrasound treatment condition were planned on the basis of effects sizes observed preliminary experiments and taking into account potential attrition. For blood brain barrier experiments n=4-5 mice were prepared per group on the basis of expected effect sizes from preliminary data, to account for potential difficulties with fluorescent dextran injection or perfusion.
Data exclusions	For the in vitro experiments in primary neurons, expression of the channel, as assessed by immunostaining, was established as a criteria to include/exclude the data. Thus rounds of neuron experiments where no channel could be detected were excluded from calcium imaging and pharmacology figures.<br><br>For in vivo EMG experiments, all data collected from GFP and 4E13 hsTRPA1-injected mice that survived were included in the study. However, a concurrent cohort that had been injected with 2E13 hsTRPA1 was excluded due to weak hsTRPA1 expression and inconclusive results. All EMG traces were included in analysis excepting in cases in which electrical interference precluded meaningful analysis of the response latency and duration. No data were excluded from c-fos analysis. For the blood brain barrier experiment, 1 naive mouse and 1 ultrasound-treated mouse were excluded due to poor perfusion.
Replication	Each in vitro experiment presented in the paper was repeated multiple times. While a few rounds of neuron culture were excluded due too expression problems (see above , Data exclusions) the data presented in the paper is drawn for at least 2 independent experiments with neurons plated from different embryos and multiple coverslips imaged. The same approach was applied for dissociated neuronal culture for patch-clamp electrophysiology.<br><br>The mouse EMG data presented within were only performed once as reported virus titers. However, a previous study used 1E13 of AAV9-hsyn-DIO-TRPA1, in which the TRPA1 virus was not tagged. EMG responses were not as robust relative to those observed in the 4E13 AAV9-hsyn-DIO-TRPA1-myc study presented herein, but otherwise showed significantly more EMG responses relative to GFP-only control (% EMG responses to 100 ms ultrasound (all pressures): GFP 10.9 +/- 4.18 vs hsTRPA1 39.3 +/- 9.19, p=0.0003 by Mann-Whitney U test). However, these data are not presented because expression of hsTRPA1 could not be reliably detected without the myc tag.<br><br>All experiments were successfully replicated
Randomization	For in vivo studies, group-housed mice were randomly assigned to GFP control or TRPA1-myc injected groups within cages, thereby ensuring equal balance across slight variations in date of birth and sex.
Blinding	Investigators were not blinded for in vitro experiments. Our candidate screen was performed blind and the data analysis was automated. We feel that this allowed us to discover the underlying mechanisms in an unbiased fashion.<br>Investigators were blinded throughout data collection, imaging and data analysis of all in vivo experiments.

Reporting for specific materials, systems and methods

We require information from authors about some types of materials, experimental systems and methods used in many studies. Here, indicate whether each material, system or method listed is relevant to your study. If you are not sure if a list item applies to your research, read the appropriate section before selecting a response.

Materials & experimental systems

n/a	Involved in the study
☐	☒ Antibodies
☐	☒ Eukaryotic cell lines
☒	☐ Palaeontology and archaeology
☐	☒ Animals and other organisms
☒	☐ Human research participants
☒	☐ Clinical data
☒	☐ Dual use research of concern

Methods

n/a	Involved in the study
☒	☐ ChIP-seq
☒	☐ Flow cytometry
☒	☐ MRI-based neuroimaging

Antibodies

Antibodies used

TRPA1 mouse monoclonal clone C5,Santa Cruz Laboratories, sc-376495 Lot #J0419, Immunofluorescence
myc rabbit monoclonal Clone 71D10, Cell Signaling Tech, 2278S, Lot #6, Immunofluorescence
Alexa Fluor™ 488 Phalloidin, Thermo Fisher, A12379, Lot #2129460, Immunofluorescence
alpha-tubulin rat monoclonal Clone YOL1/34, Sigma Aldrich, CBL270-I, Lot# 3011478, Immunofluorescence
c-Fos rabbit polyclonal, Encor Biotechnology, RCPA-cfos, Lot #170-080216, Immunofluorescence
NeuN guinea pig polyclonal, Synaptic Systems, 226004, Lot #2-23, Immunofluorescence
GFP chicken polyclonal, Aves, GFP-1010, Lot #GFP879484, Immunofluorescence
Biotinylated donkey anti-rabbt. Jackson Immunoresearch, 711-065-152, Lot # 144433, Immunofluorescence

Validation

TRPA1 has been validated by Virk et al, (https://pubmed.ncbi.nlm.nih.gov/31811235/). Furthemore, we only observed TRPA1 signal in cells that had been transfected with the bicistronic hsTRPA1, dtom construct, specifically in cells that were confirmed to express dtom (Figure 1d).

Myc signal was detected only in hsTRPA1-myc injected mouse cortical neurons. No signal was detected in GFP-only injected animals. All myc signal was detected within neurons that co-expressed GFP, suggesting successful transduction with the viral vector (data available upon request).

Use of phallodin as a marker to detect actin is discussed in detail by Kumari et al, 2020 (https://www.embopress.org/doi/10.15252/embj.2019104006)

Alpha-tubulin antibody validation information can be found at the manufacturer's website, (https://www.sigmaaldrich.com/catalog/product/mm/cbl270-i?lang=en&region=US) and in the following two publications: A representative lot immunostained fixed NIH/3T3 cells as well as yeast spheroplasts and nuclei preparations (Kilmartin, J.V., et al. (1982). J. Cell Biol. 93(3):576-582). and A representative lot immunostained microtubules of fixed Dictyostelium (amoeba) as well as isolated and stabilized amoeba cytoskeleton (Kilmartin, J.V., et al. (1982). J. Cell Biol. 93(3):576-582).

c-Fos antibody product information and validation can be found at the manufacturer's website: https://encorbio.com/product/rpca-c-fos/

NeuN antibody validation information can be found at the manufacturer's website: https://sysy.com/product/266004

GFP antibody validation information can be found at the manufacturer's website: https://www.aveslabs.com/products/green-fluorescent-protein-gfp-antibody. We also observed GFP immunoreactivity only in brain sections from mice that had been injected with AAV9-hSyn-DIO-GFP, and only in regions near the injection site in the expected neurons. Data available upon request.

Eukaryotic cell lines

Policy information about cell lines

Cell line source(s)

HEK293 (ATCC® CRL-1573)
HEK cells expressing human αvß3 integrin (Patel et al, 2013) were provided by Dan Gibbs, UCSD.
Ex-HEK (HEK cells expressing Nav1.3 and Kir2.1, Park et al, 2013) were acquired from ATCC CRL-3269

Authentication

None of the cell lines were authenticated

Mycoplasma contamination

PlasmoTest kit from Invitrogen (rep-pt1) was used to confirm the absence of mycoplasma contamination in all the cell lines used.

Commonly misidentified lines
(See ICLAC register)

No commonly misidentified cell lines were used in the study

Animals and other organisms

Policy information about studies involving animals; ARRIVE guidelines recommended for reporting animal research

Laboratory animals	A total of 50 mice were used in our studies.<br>For mouse neuronal cultures: WT E18 embryos were collected from timed-pregnant C57Bl/6 mice (JAX# 000664) (n=3 pregnant mice total). TRPA1-/- E18 embryos were collected from a TRPA1-/- (JAX #006401) female bred with a TRPA1-/- male mouse (n=2 pregnant mice total).<br>Hydrophone measurements were taken in 1 18 week-old male Npr3-cre mouse , 1 12 week-old female Npr3-cre mouse and 1 12-week-old Bl/6 male mouse (n=3 mice total). Brain and DRG tissue for Basescope analysis was taken from an 11 week-old female C57Bl6/J mouse (JAX# 000664), a 20 week-old female TRPA1 -/- mouse (JAX #006401) and an E18 embryo taken from a timed-pregnant C57Bl/6 mouse (JAX# 000664). Homozygous Npr3-cre mice (JAX# 031333) received cortical viral injections between 12-20 weeks of age. EMG data were collected between 2-4 weeks after injection. Data presented are from n=2 males and n=3 females for GFP and n=1 males and n=4 females for hsTRPA1-injected mice. (n=10 mice total). For rotarod and c-fos experiments, homozygous Npr3-cre mice (JAX# 031333) received cortical viral injections between 10-20 weeks of age. Rotarod data were collected 2 weeks after injection. c-fos data were collected 6 weeks after injection. Delays in c-fos data collection were in part due to the Covid-19 shutdowns. N=5 males and n=2 females were included in GFP and hsTRPA1-injected groups. (n=14 mice total). For blood brain barrier experiments age-matched Balb/c mice (JAX # BALB/cJ/000651) were used in experiments at 9 weeks of age. All groups included n=2 male and n=2 female mice, excepting the stab wound condition, which included n=2 male and n=3 female mice. (n=17 mice total).<br>50 mice
Wild animals	This study did not involve wild animals.
Field-collected samples	This study did not involve samples collected from the field.
Ethics oversight	Animals were group housed in an American Association for the Accreditation of Laboratory Animal Care approved vivarium, and all protocols were approved by the Institutional Animal Care and Use Committee of the Salk Institute for Biological Studies.

Note that full information on the approval of the study protocol must also be provided in the manuscript.
