# Supplementary material for: Sonogenetic control of mammalian cells using exogenous Transient Receptor Potential A1 channels
Source: Nat Commun. 2022 Feb 9;13:600. doi: 10.1038/s41467-022-28205-y (PMC8828769; doi:10.1038/s41467-022-28205-y)
Supplement: Supplementary file 4 — Description of Additional Supplementary Files [file 41467_2022_28205_MOESM4_ESM.pdf]

Title: Supplementary Movie 1.
Description: *hs*TRPA1-HEK cells respond to single ultrasound pulses at 7MHz. Representative response to a single 100ms ultrasound pulse in *hs*TRPA1-expressing HEK cells. Color scale: red, high GCaMP6f ΔF/F signal, blue, low GCaMP6f ΔF/F. Scale bar 20 μm. Time scale is in seconds. Stimulation parameters: 100ms 2.5 MPa 7MHz delivered at t = 20 s.

Title: Supplementary Movie 2.
Description: HEK cells are normally insensitive to ultrasound at 7MHz. Lack of response to a single 100ms ultrasound pulse in dTomato-expressing HEK cells, as assessed by calcium imaging. Color scale: red, high GCaMP6f ΔF/F signal, blue, low GCaMP6f ΔF/F. Scale bar 20 μm. Time scale is in seconds. Stimulation parameters: 100ms 2.5 MPa 7MHz delivered at t = 20 s.

Title: Supplementary Movie 3.
Description: *hs*TRPA1 increases sensitivity to 7MHz ultrasound in mouse primary neurons *in vitro.* Representative response to a single 100ms ultrasound pulse in mouse cortical primary neurons (DIV10-11) that were infected with AAV9-hSyn-DIO-*hs*TRPA1 and AAV9-Cre. Color scale: red, high GCaMP6f ΔF/F signal, blue, low GCaMP6f ΔF/F. Scale bar 20 μm. Time scale is in seconds. Stimulation parameters: 100 ms 2.5 MPa 7 MHz delivered at t = 20 s.

Title: Supplementary Movie 4.
Description: Mouse primary neurons intrinsically show modest responses to 7MHz ultrasound. Representative response to a single 100ms ultrasound pulse in control mouse cortical primary neurons (DIV10-11) that were infected with only AAV9-Cre. Color scale: red, high GCaMP6f ΔF/F signal, blue, low GCaMP6f ΔF/F. Scale bar 20 μm. Time scale is in seconds. Stimulation parameters: 100 ms 2.5 MPa 7 MHz delivered at t = 20s.

Title: Supplementary Movie 5.
Description: *hs*TRPA1 expression allows robust repeated ultrasound stimulation in mouse primary neurons *in vitro*. This video shows a representative response to repetitive100ms ultrasound pulses delivered every 10s (0.1Hz) in mouse cortical primary neurons (DIV10-11) that were infected with AAV9-hSyn-DIO-*hs*TRPA1 and AAV9-Cre. Color scale: red, high GCaMP6f ΔF/F signal, blue, low GCaMP6f ΔF/F. Scale bar 20 μm. Time scale is in seconds. Stimulation parameters: 100 ms 2.5 MPa 7MHz.

Title: Supplementary Movie 6.
Description: *hs*TRPA1 expression allows for ultrasound-evoked contralateral hindlimb movement. This video shows a representative right hindlimb (contralateral to injection site) response to repetitive 0.88 MPa ultrasound delivered for 10 ms or 100 ms in an Npr3-cre mouse expressing *hs*TRPA1 in the left motor cortex. The red LED and “Ultrasound” text indicate when ultrasound is on. No left limb movements were observed.
